# Development and psychometric properties of Chinese social emotional competence measurements

**DOI:** 10.3389/fpsyg.2025.1574923

**Published:** 2025-07-31

**Authors:** Hongfei Tan, Zhen-Dong Wang, Xia Xiao, Zhaoyue Jing, Keying Chen, Xiu Yao

**Affiliations:** ^1^School of Traditional Chinese Medicine, Shanghai University of Traditional Chinese Medicine, Shanghai, China; ^2^Penguin Qiqi Art with SEL Research Centre, Beijing, China; ^3^Specialized Community Services, CBI Health Group, Calgary, AB, Canada; ^4^The Department of Computer and Mathematical Sciences, University of Toronto Scarborough, Toronto, ON, Canada

**Keywords:** social-emotional learning (SEL), social and emotional competence, SEL measurement, SEL scale, Chinese college students

## Abstract

**Introduction:**

Social-emotional competence (SEC) plays a critical role in the personal and academic development of university students. However, there is a lack of culturally appropriate tools to assess SEC in Chinese populations. This study aimed to develop and validate Chinese versions of two established SEC measurements: the Social Emotional Competence Questionnaire (C-SECQ) and the Social-Emotional Learning Scale (C-SELS).

**Methods:**

Two studies were conducted using independent samples of Chinese university students. Study 1 (*N* = 195) involved preliminary psychometric evaluation, while Study 2 (*N* = 540) provided an independent validation. Both internal consistency and construct validity were examined for the C-SECQ. For the C-SELS, exploratory factor analysis (EFA) was conducted in Study 1, and the resulting model was tested in Study 2 for convergent and discriminant validity.

**Results:**

The C-SECQ demonstrated high internal consistency and strong construct validity across both samples, supporting its suitability for use in the Chinese context. In contrast, EFA of the C-SELS revealed a new three-factor structure: Emotional and Social Awareness, Goal Setting and Problem Solving, and Emotional Regulation and Responsibility. However, this revised model showed limited convergent and discriminant validity in Study 2, indicating insufficient psychometric support.

**Discussion:**

These findings support the C-SECQ as a reliable and valid tool for assessing SEC among Chinese university students. The study also highlights the challenges in adapting and validating the C-SELS, emphasizing the need for further refinement and cross-cultural validation. Overall, this research contributes to the development of context-appropriate SEL assessment tools in Chinese educational settings.

## Introduction

Social and Emotional Learning (SEL, also referred to as ‘social-emotional learning’ or ‘socio-emotional learning’) is an educational approach that integrates the development of social and emotional competencies into school curricula, which has been widespread in various countries because of the increasing concerns of students’ mental health and social skills (Borowski, 2019; Durlak et al., 2011).

In the United States, the Collaborative for Academic Social and Emotional Learning (CASEL) has been a leading force in promoting SEL, advocating that all children should be equipped with the ability to become lifelong learners who are self-aware, empathetic, and capable of making responsible decisions. CASEL’s framework is built upon five core competencies: self-awareness, self-management, social awareness, relationship skills, and responsible decision-making (Borowski, 2019).

The body of past research on SEL can be broadly categorized into two streams. Firstly, many studies focus on the cross-cultural analyses of the dimensions of SEL, examining how different cultural contexts shape the understanding and implementation of SEL programs. For example, in the United Kingdom, Department for Education and Skills (DES) started the program Social and Emotional Aspects of Learning (SEAL), and it focuses on Self-awareness, Self-regulation, Motivation, Empathy and Social Skills, while overlapping with CASEL’s dimensions, places additional emphasis on self-regulation, motivation, and empathy, reflecting the unique cultural and educational priorities in the U.K. (Humphrey et al., 2009). Yu and Jiang (2017) put forward a six-dimensional SEL framework based on their practice in China that is highly consistent with CASEL framework, comprising self-cognition (knowing about and reflecting on one’s own feelings), self-management (adjusting one’s own emotion to assist with tasks at hand), cognition of others (understanding others’ feelings and perspectives), management of others (dealing with emotional issues in interpersonal relationships), cognition of the collective (understanding the rules, norms and values of the collective and its perspective), and management of the collective (building a sense of belonging to the collective).

Secondly, there are also some empirical studies testing the effectiveness of the current SEL programs. These studies, particularly in classroom settings, have demonstrated that SEL interventions can significantly enhance students’ academic performance, reduce substance use, and mitigate aggression and antisocial behavior (Weissberg and O’Brien, 2004).

In both streams of study, well-developed and reliable measures of social–emotional competence had been prevalent. Prior to the development of SEL scales, existing scales of relevant SEL dimensions were used to assess social–emotional competence. Considering these scales were predominantly designed for young children, they generally included self-reports as well as forms for teachers, parents and peers to avoid potential bias from self-reports, and they often put more emphasis on academic performance.

For example, Merrell (1993) developed School Social Behavior Scales (SSBS), which measures social competence and antisocial behaviors by behavior rating scales. The SSBS showed strong relevance to the dimensions of SEL, particularly in areas such as interpersonal skills and self-management skills. A similar framework can also be seen in Social Skills Rating System (SSRS), but it exhibited a lower interrater reliability, largely due to its focus on students in kindergarten through third grade, with assessments primarily based on teacher and parent reports (Elliott et al., 1988). The Behavior Assessment System for Children (BASC) covered a wider range of age group from preschool students to adolescence, and focused on adaptive and problem behaviors (Sandoval and Echandia, 1994). In addition to school settings, the BASC also uses parent forms to evaluate children’s behaviors in home and community settings. Difference in reliability coefficients were observed across different age groups, with assessments for adolescents demonstrating higher reliability.

As the understanding of SEL deepens, an increasing number of psychological scales that are designed based on SEL dimensions has been developed, and most of the assessments demonstrate high level of consistency with the CASEL framework (Martinez-Yarza et al., 2023). One such example is the Social Skills Improvement System Social Emotional Learning Edition Rating Forms (SSIS SEL RF), which was constructed based on the notion of “social skills” from the previous iterations of SSRS and SSIS. These skills are well suited to the CASEL five domains of SEL, particularly within the Parent and Student Forms. The Teacher Form, however, introduced an additional sixth domain focusing on “academic competence” (Gresham et al., 2020). Further contribution to the field, Zhou and Ee (2012) designed Social Emotional Competence Questionnaire (SECQ), a 25-item scale based on CASEL’s SEL domains, showed high reliability and validity when tested among secondary school students in Singapore, underscoring its potential for cross-cultural application in SEL study. Similarly, Kneidel (2022) developed the Student Engagement in Social-Emotional Learning Skills (SE-SELS) to provide a reliable and valid tool for measuring the outcomes in school-based SEL interventions. The SE-SELS, which aligns with the CASEL five dimensions, showed a high level of internal reliability (*α* = 0.90). However, its external validity may be limited, as the study’s participants were drawn from a single school. Coryn et al. (2009) also contributed to the field with the development of the Social-Emotional Learning Scale (SELS), a 20-item scale based on CASEL domains. Although exploratory factor analysis (EFA) revealed a three-factor model—Task Articulation (TA), Peer Relationships (PR), and Self-Regulation (SR)—these factors closely correspond to the original five dimensions. However, the estimated interfactor correlations indicated weaker discriminant validity, highlighting the need for further factor analysis.

In recent years, SEL has garnered increasing attention in China, driven by a growing recognition of the pressing need to address students’ mental health challenges, particularly those related to social and emotional skills. A large proportion of adolescents in China reported depressive and anxiety symptoms (Zhou et al., 2020). The rising incidence of anxiety, depression, and emotional distress among Chinese students highlights the necessity of educational approaches that extend beyond mere academic performance (Fu and Zhang, 2023). Simultaneously, educators and policymakers in China are increasingly questioning the traditional education model that places excessive emphasis on academic exam scores. This narrow focus is being reconsidered as inadequate for preparing students to navigate a rapidly changing society that demands not only academic excellence but also critical thinking, collaboration, and emotional intelligence. As a result, there is a growing interest in integrating SEL into the educational system to cultivate these vital competencies (Shi and Li, 2023). In China, a school-based pilot SEL program has been proven effective in reducing elementary school students’ psychosocial difficulties (Li and Hesketh, 2024). However, in terms of integrating SEL into the Chinese context, both researchers and educators are currently in the early stages, which indicates the necessity of the localization (Liu X., 2021). Therefore, it is essential to develop assessment tools that are culturally relevant and tailored to the unique social and cultural dynamics of Chinese society.

Most existing psychological scales for SEL are developed in North America, highlighting the need for cross-cultural studies that adapt these tools to other languages and cultural contexts (Hayashi et al., 2022). Although several SEL scales have been translated into Chinese and utilized in applied studies. For instance, Liu J. (2021) developed a Chinese version of Social Skills Improvement System Social Emotional Learning Edition (SSIS-SEL), which is based on CASEL’s five-factor model. This scale demonstrated acceptable goodness-of-fit and high reliability in the Chinese context. However, during our preliminary study, feedback indicated that some items on the scale were ambiguous and unclear, suggesting potential issues in the translation process. The availability of high-quality Chinese SEL measurements remains limited, despite the growing mental health challenges faced by students across various age groups in China, such as students in colleges and universities (Gao et al., 2020; Wang et al., 2021). Thus, it is crucial to create reliable psychological scales tailored to the Chinese cultural context to assess individuals’ social–emotional competence effectively.

This study aims to address this gap by developing and evaluating the psychometric properties of Chinese versions of two established SEL instruments: the Chinese version of Social Emotional Competence Questionnaire (C-SECQ) and the Chinese version of Social-Emotional Learning Scale (C-SELS). The selection of these specific instruments was guided by several considerations. According to a systematic review by Martinez-Yarza et al. (2023), the SECQ is a comprehensive measure assessing all five core CASEL domains and had not yet been validated in a Chinese version. The SELS, while originally presenting a three-factor structure (Task Articulation, Peer Relationships, and Self-Regulation), also demonstrates strong conceptual links to the five CASEL domains. For example, Task Articulation relates to responsible decision-making, Peer Relationships to social awareness and relationship skills, and Self-Regulation to self-awareness and self-management. We hypothesized that these two scales, while distinct in their original factor structures, could both contribute valuable insights into social–emotional competence within the Chinese context, potentially capturing different facets of the overarching construct. A key objective was to explore their adaptability and utility for Chinese university students.

Furthermore, much of the current SEL research has concentrated on school settings and younger children, leaving a gap in understanding how SEL theories and practices might apply to other age groups and diverse environments (Mahoney et al., 2021). Therefore, the current study aims to address this gap by developing Chinese-language psychological scales that systematically measure social–emotional competence through reliable self-reports from adolescents and adults. Additionally, the psychometric properties of these newly developed scales will be thoroughly evaluated.

Accordingly, we adopted a two-study design: Study 1 provides a preliminary psychometric appraisal of the translated instruments, whereas Study 2 cross-validates the revised factor structure with an independent, larger sample. Together, these complementary studies offer an initial yet rigorous assessment of the scales’ suitability for use with Chinese university students.

## Study 1: Preliminary psychometric analysis of C-SECQ and C-SELS

A preliminary study was conducted to explore the psychometric properties of two social–emotional competence measurements, C-SECQ and C-SELS, in Chinese context.

## Methods

### Participants

A total of 236 participants were initially recruited for the study. Two attention-check items were included to avoid carelessness, and participants who failed any of the two items were excluded from data analysis. Consequently, 195 valid participants, aging 17 and above (*M* = 19.09, *SD* = 1.11), with 69 males and 126 females, were available for analysis. All participants were native speakers of Mandarin Chinese and were enrolled in an Introductory Psychology course at a university in Shanghai. None of the participants were psychology majors, and all reported having very limited prior background knowledge of psychology, ensuring a comparable baseline understanding of the questionnaire items.

### Materials and procedures

Two Chinese versions of Social Emotional Learning scales were utilized in this study.

**Chinese version of Social Emotional Competence Questionnaire (C-SECQ):** Adapted from Zhou and Ee (2012), this 25-item self-report scale measures SEL skills using a 6-point Likert scale (1 = *Not at all True of me* to 6 = *Very True of me*). It assesses CASEL’s five domains: self-awareness (SA, e.g., “I know what I am thinking and doing”), self-management (e.g., “I can stay calm in stressful situations”), social awareness (SoA, e.g., “It is easy for me to understand why people feel the way they do”), relationship skills (RS, e.g., “I will always apologize when I hurt my friend unintentionally”), and responsible decision-making (RDM, e.g., “When making decisions, I take into account the consequences of my actions”).

**Chinese version of Social-Emotional Learning Scale (C-SELS):** Adapted from Coryn et al. (2009), this 20-item self-report scale uses a 5-point Likert scale (1 = *Strongly Disagree* to 5 = *Strongly Agree*). The original English version is organized into three dimensions: Task Articulation (TA; related to responsible decision-making, e.g., “Understand situations that cause me to feel happy, sad, angry, or frustrated”), Peer Relationships (PR; focusing on social awareness and relationship skills, e.g., “Understand the feelings expressed by others”), and Self-Regulation (SR; pertaining to self-awareness and self-management, e.g., “Understand that I am responsible for my own actions”).

**The translation of these scales followed a rigorous three-step process:** (1) Initial Translation: The original English versions of the scales were directly translated into Chinese, with minor adjustments made to enhance clarity without altering the meaning of the items. (2) Back-Translation: Two individuals, fluent in English but without psychology backgrounds, independently back-translated the Chinese versions into English. (3) Comparison and Refinement: The back-translated versions were compared with the original English scales. Discrepancies and potential misunderstandings were addressed, resulting in the final Chinese versions.

Participants provided informed consent and completed a demographics form (age, gender, ethnicity) online. The survey was administered anonymously, with assurances that no information would be disclosed to third parties. Participants then completed the C-SECQ and C-SELS. A debriefing page was provided at the end.

### Data analysis

Data analysis was conducted using IBM SPSS 26.0 and Mplus 8.3. Internal consistency reliability for each scale and its subscales was assessed using Cronbach’s alpha and McDonald’s omega (*ω*). Confirmatory Factor Analysis (CFA) using Maximum Likelihood (ML) estimation was conducted to test the fit of the original factor structures of the C-SECQ and C-SELS. Likert-scale items were treated as continuous variables for ML estimation, a common practice for scales with five or more categories when data do not severely violate normality assumptions (Bollen and Long, 1992; Rhemtulla et al., 2012). Model fit was evaluated using the chi-square statistic (chi-square), degrees of freedom (df), chi-square/df ratio, Root Mean Square Error of Approximation (RMSEA), Comparative Fit Index (CFI), and Tucker-Lewis Index (TLI). CFI/TLI values ≥0.90 are generally considered acceptable, with values ≥0.95 indicating good fit (Hu and Bentler, 1999). RMSEA values ≤0.08 suggest acceptable fit, and ≤0.06 suggest good fit (Browne and Cudeck, 1992). Exploratory Factor Analysis (EFA) with Varimax rotation and Kaiser Normalization was conducted if the initial CFA model fit was poor, to explore alternative factor structures. Factors were considered for extraction if eigenvalues were greater than 1, and the cumulative variance explained was substantial (e.g., > 50–60%), also considering the scree plot. Convergent and discriminant validity were further assessed using Average Variance Extracted (AVE) and Maximum Shared Variance (MSV). AVE > 0.50 is desirable for convergent validity, and AVE > MSV supports discriminant validity (Fornell and Larcker, 1981). The H-index was calculated as an indicator of construct replicability (Hancock and Mueller, 2006). No missing data were present in the final samples.

## Results

The details of descriptive statistics of each item in C-SECQ and C-SELS have been listed in Supplementary Tables S1, S2. To provide additional evidence of convergent validity between the two instruments, the correlations among their respective dimensions were examined (see Supplementary Table S3).

As the primary goal of this research is the adaptation of established instruments, our analytical strategy prioritized a theory-driven approach. Confirmatory Factor Analyses (CFA) were conducted to formally test whether the original, theoretically-derived factor structures of the C-SECQ and C-SELS were applicable to Chinese university student sample. This initial CFA serves as a critical test of the original models’ cross-cultural viability. Following this, for any instrument that did not demonstrate an adequate model fit via CFA, Exploratory Factor Analysis (EFA) was subsequently applied. The purpose of the EFA was not to validate a model, but rather to explore the data for an alternative, empirically-driven factor structure that could better represent the construct in our sample and form a new hypothesis for validation.

### Initial test of the original factor structures: confirmatory factor analysis

**Figure 1:**
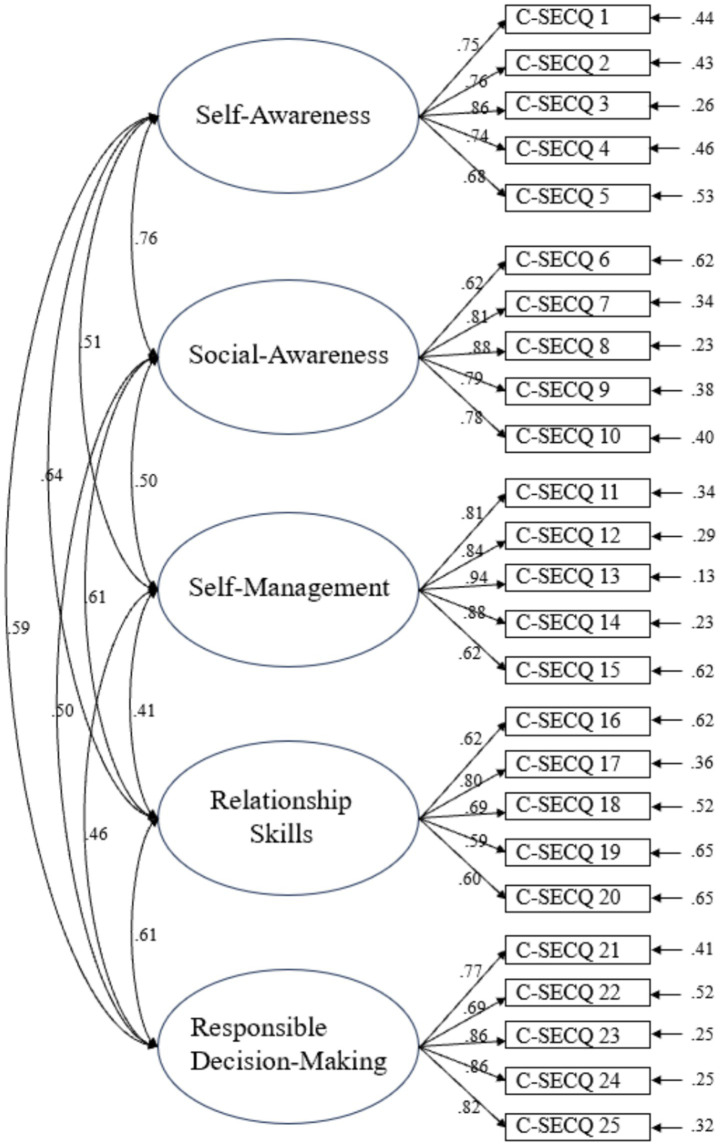
The diagram of CFA results for C-SECQ (*N* = 195). Factor labels derived from original scale structure.

In terms of C-SECQ, CFA results for the original five-factor model indicated a marginally acceptable model fit: χ^2^ = 503.43 (*df* = 265, *p* < 0.001), χ^2^/*df* = 1.90, RMSEA = 0.068, CFI = 0.89, TLI = 0.88. While RMSEA was acceptable, the CFI and TLI values were slightly below the conventional 0.90 threshold, suggesting a suboptimal fit. Figure 1 illustrates the CFA path diagram. Most factor loadings exceeded 0.60, except for item C-SECQ 19.

**Figure 2:**
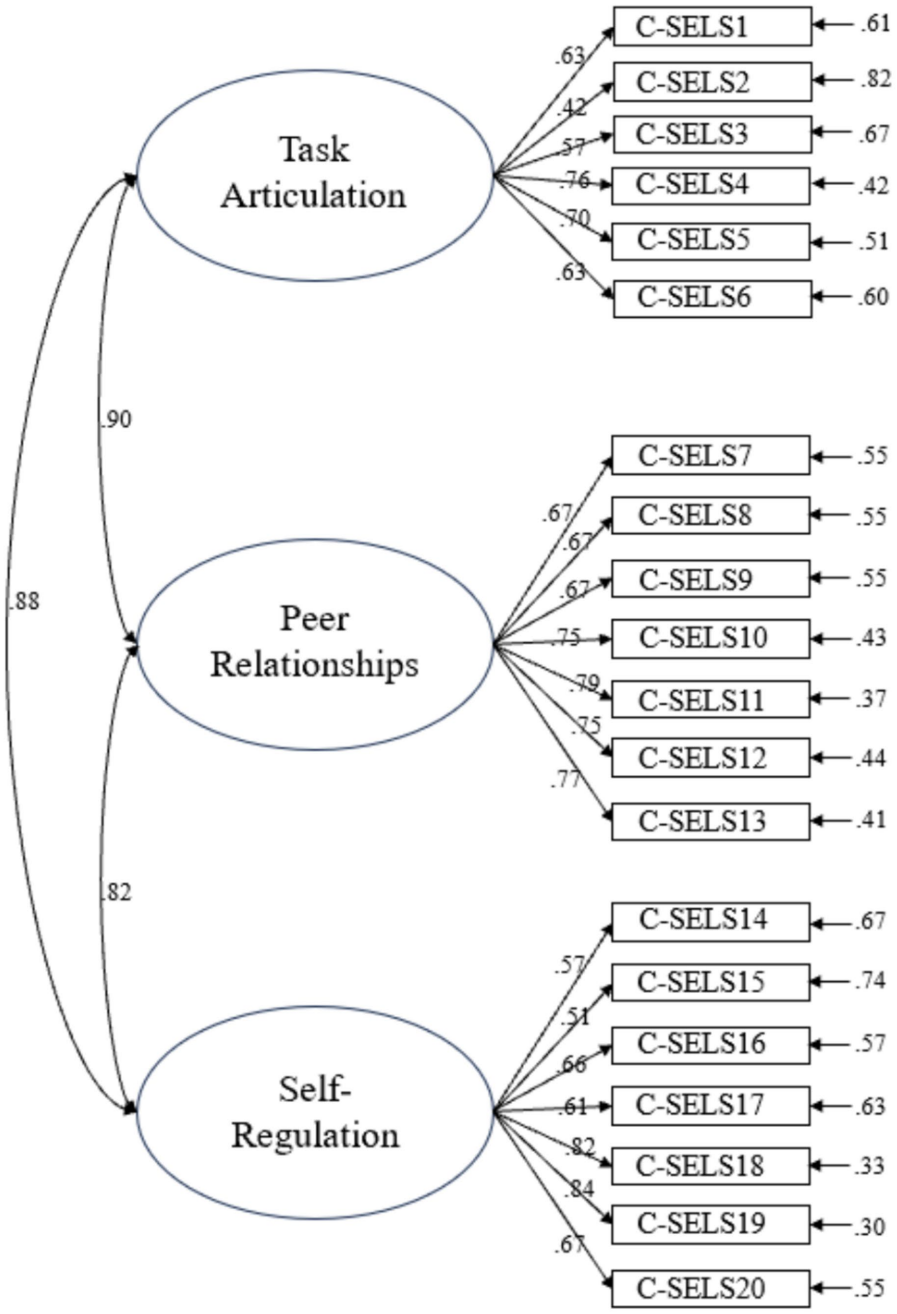
The diagram of CFA results for C-SELS (*N* = 195). Factor labels derived from original scale structure.

In addition, CFA of the original three-factor C-SELS model indicated a poor fit: χ^2^ = 525.79 (*df* = 167, *p* < 0.001), χ*^2^/df* = 3.15, *RMSEA* = 0.11, *CFI* = 0.83, *TLI* = 0.80, suggesting potential structural issues within the factors. The results of CFA are also shown in Figure 2. Furthermore, an examination of the original three-factor model’s construct validity revealed poor discriminant validity, with AVE values being lower than MSV values for all factors (e.g., Emotional and Social Awareness: AVE = 0.45, MSV = 0.84), indicating substantial overlap and warranting model refinement.

#### Exploration of alternative factor structures: exploratory factor analysis

**Figure 3:**
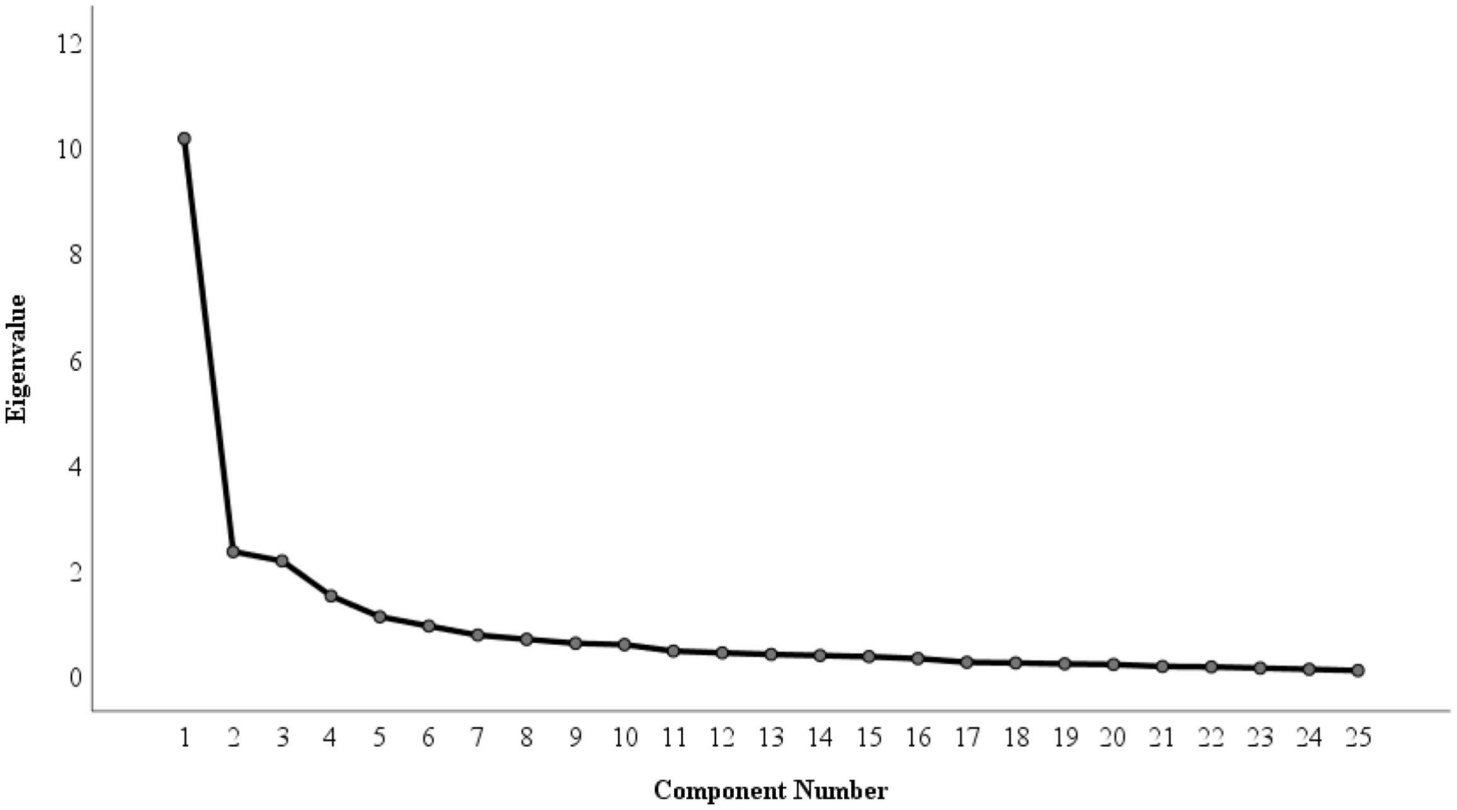
The scree plot of the EFA results of C-SECQ. The factors are extracted if the eigenvalues are greater than 1.

Furthermore, considering the CFA results of C-SECQ suggested a suboptimal fit, EFA (Varimax rotation, Kaiser Normalization) was conducted in C-SECQ. Factors were extracted if eigenvalues > 1 and cumulative variance explained >0.60. The scree plot has been shown in Figure 3. The EFA results (Table 1) largely mirrored the original five-factor structure (Cumulative Variance Explained [CV] = 69.224%). One notable exception was Item 5 (“*I can read people’s faces when they are angry*”), which loaded more strongly on Social Awareness (0.65) than its original Self-Awareness dimension, suggesting a potential cultural nuance in its interpretation.

**Table 1:** Rotated component matrix of EFA results of C-SECQ (*N* = 195).

Items	Self-awareness	Social awareness	Self-management	Relationship skills	Responsible decision-making
C-SECQ1	**0.72**				
C-SECQ2	**0.69**				
C-SECQ3	**0.76**				
C-SECQ4	**0.66**				
C-SECQ5		**0.65**			
C-SECQ6		**0.59**			
C-SECQ7		**0.78**			
C-SECQ8		**0.84**			
C-SECQ9		**0.69**			
C-SECQ10		**0.69**			
C-SECQ11			**0.80**		
C-SECQ12			**0.85**		
C-SECQ13			**0.87**		
C-SECQ14			**0.85**		
C-SECQ15			**0.62**		
C-SECQ16				**0.68**	
C-SECQ17				**0.69**	
C-SECQ18				**0.74**	
C-SECQ19				**0.67**	
C-SECQ20				**0.50**	
C-SECQ21					**0.79**
C-SECQ22					**0.70**
C-SECQ23					**0.86**
C-SECQ24					**0.80**
C-SECQ25					**0.75**

“C-SECQ1” refers to the item 1 from C-SECQ Scale. Factor labels derived from original scale structure with item 5 moved to Social Awareness. The bolded values are factor loadings for each item in the corresponding dimension. Loadings <0.40 are suppressed for clarity. Cumulative variance explained CV = 69.224%.

**Figure 4:**
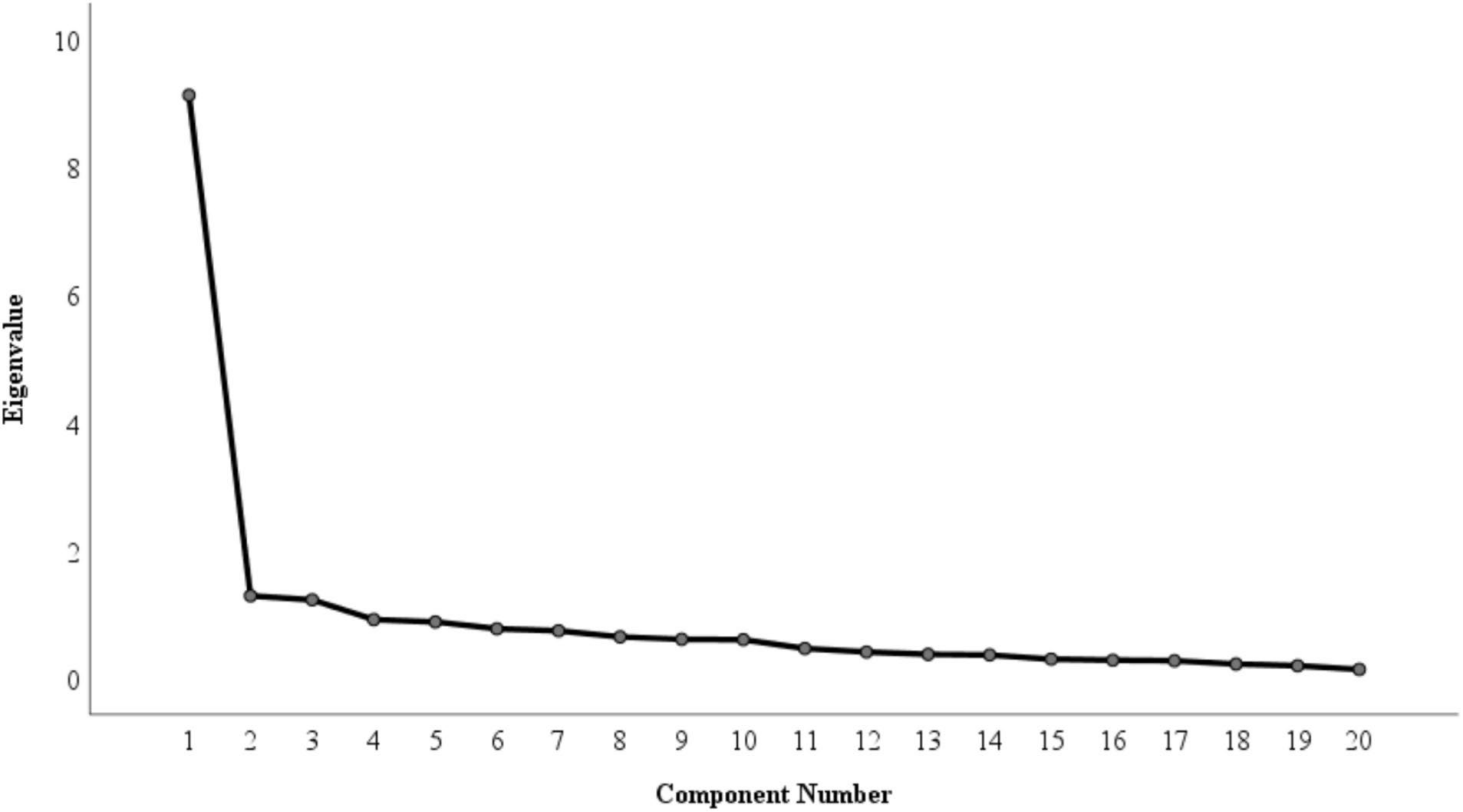
The scree plot of the EFA results of C-SELS. The factors are extracted if the eigenvalues are greater than 1.

Similarly, given the poor fit reported in C-SELS, EFA (Varimax rotation, Kaiser Normalization) was conducted on the C-SELS items. The scree plot (Figure 4) and eigenvalue > 1 criterion suggested a three-factor solution.

This EFA (CV = 58.239%, approaching the 60% threshold) yielded a different factor structure from the original (Table 2).

**Table 2:** Rotated component matrix of EFA results of C-SELS (*N* = 195).

Items	Factor 1	Factor 2	Factor 3
C-SELS1			**0.64**
C-SELS2	**0.42**		
C-SELS3	**0.55**		
C-SELS4	**0.48**		
C-SELS5		**0.76**	
C-SELS6	**0.58**		
C-SELS7			**0.56**
C-SELS8	**0.47**		
C-SELS9	**0.54**		
C-SELS10	**0.83**		
C-SELS11	**0.82**		
C-SELS12	**0.60**		
C-SELS13	**0.62**		
C-SELS14			**0.82**
C-SELS15			**0.72**
C-SELS16			**0.56**
C-SELS17		**0.64**	
C-SELS18		**0.77**	
C-SELS19		**0.81**	
C-SELS20		**0.57**	

“C-SELS 1” refers to the item 1 from C-SELS. The bolded values are factor loadings for each item in corresponding dimension. Loadings <0.40 are suppressed for clarity. Cumulative variance explained CV = 58.239%.

The newly extracted factors were named by inductively deriving the thematic content implied by the semantics of their constituent items: Factor 1 (Emotional and Social Awareness), Factor 2 (Goal Setting and Problem Solving), and Factor 3 (Emotional Regulation and Responsibility). These categorizations differ from the original three dimensions (Task Articulation, Peer Relationships, and Self-Regulation), reflecting cultural differences in how these constructs are understood. The definitions of these newly recognized factors of C-SELS are: (1) Emotional and Social Awareness refers to the ability to recognize and understand one’s own emotions and those of others, as well as to interpret social cues and respond appropriately in interpersonal interactions. (2) Goal Setting and Problem Solving reflects the capacity to establish clear objectives, develop strategic plans, and systematically overcome obstacles to achieve desired outcomes. (3) Emotional Regulation and Responsibility involves managing emotional responses, maintaining self-control under stress, and taking accountability for one’s actions and decisions.

### Internal consistency and construct validity of the emergent structures

After conducting EFA, C-SECQ with new factor structure demonstrated high overall internal consistency (Cronbach’s *α* = 0.94). Cronbach’s alphas for five dimensions were: Self-awareness (0.86), Self-management (0.88), Social Awareness (0.90), Relationship skills (0.79), and Responsible decision-making (0.90).

As shown in Table 3, McDonald’s *ω* for the total C-SECQ scale indicated excellent internal consistency. AVE values for the factors ranged from 0.59 to 0.68, exceeding.50 and supporting convergent validity (Fornell and Larcker, 1981). For most factors, MSV values were lower than AVE values, supporting discriminant validity, though Self-Awareness had an MSV (0.62) close to its AVE (0.63). The construct replicability index (H) also suggested a well-defined latent construct (Hancock and Mueller, 2006).

**Table 3:** The validity psychometric indicators of C-SECQ (*N* = 195).

Factor	AVE	H	ω	MSV
Self-awareness	0.63	0.87	0.87	0.62
Social awareness	0.60	0.90	0.89	0.54
Self-management	0.68	0.91	0.90	0.27
Relationship skills	0.59	0.80	0.83	0.40
Responsible decision-making	0.65	0.90	0.90	0.37

Factor labels derived from new scale structure obtained by EFA. AVE = Average Variance Extracted; H = construct replicability index; ω = McDonald’s omega; MSV = Maximum Shared Variance. Indices were calculated based on standardized factor loadings.

In addition, the C-SELS showed high overall internal consistency (Cronbach’s α = 0.93). Based on the newly defined three factors, Cronbach’s alphas were: Emotional and Social Awareness (0.89), Goal Setting and Problem Solving (0.86), and Emotional Regulation and Responsibility (0.81). McDonald’s ω values were also high (Table 4). However, for the original three-factor model, AVE values were low relative to MSV values for all factors (e.g., Emotional and Social Awareness: AVE = 0.45, MSV = 0.84), indicating poor discriminant validity and substantial overlap, warranting model refinement.

**Table 4:** The validity psychometric indicators of C-SELS (*N* = 195).

Factor	AVE	H	ω	MSV
Emotional and social awareness	0.45	0.77	0.89	0.84
Goal setting and problem solving	0.41	0.80	0.77	0.62
Emotional regulation and responsibility	0.35	0.72	0.72	0.84

Factor labels derived from new scale structure obtained by EFA. AVE = Average Variance Extracted; H = construct replicability index; ω = McDonald’s omega; MSV = Maximum Shared Variance. Indices were calculated based on standardized factor loadings.

## Study 2: Cross-validation of C-SECQ and C-SELS

To further examine the psychometric properties of the C-SECQ, with the item 5 adjustment from Study 1 EFA, and the newly derived three-factor model of C-SELS, an independent sample validation study was conducted with a larger sample.

## Methods

### Participants

Initially, 595 undergraduate students were recruited for data collection, all of whom were independent of the sample in Study 1. After excluding those who failed either of the two attention-check items, 540 valid responses were retained for analysis. The final sample comprised 198 males (36.7%) and 342 females (63.3%), aged 16 to 25 years (*M* = 19.17, *SD* = 1.11). All participants were undergraduate students from mainland China, with Mandarin Chinese proficiency of native level. None were psychology majors.

### Materials and procedures

Participants completed demographics forms (gender, age) and then the C-SECQ (with item 5 keyed to Social Awareness) and the C-SELS (items to be tested against the new three-factor structure from Study 1). Procedures were similar to Study 1.

### Data analysis

Data analysis procedures mirrored Study 1, focusing on the revised C-SECQ structure and the new three-factor C-SELS structure. Internal consistency (Cronbach’s *α*, McDonald’s *ω*), CFA model fit (ML estimation), EFA (Varimax rotation, Kaiser Normalization), and validity indicators (AVE, MSV, H-index) were assessed. No missing data were present in the final samples.

## Results

The details of descriptive statistics of each item in C-SECQ and C-SELS have been listed in Supplementary Tables S4, S5. To further assess the construct validity of the measures, the inter-correlations among the dimensions of the revised C-SECQ and the new three-factor C-SELS were analyzed (see Supplementary Table S6).

### C-SECQ

**Figure 5:**
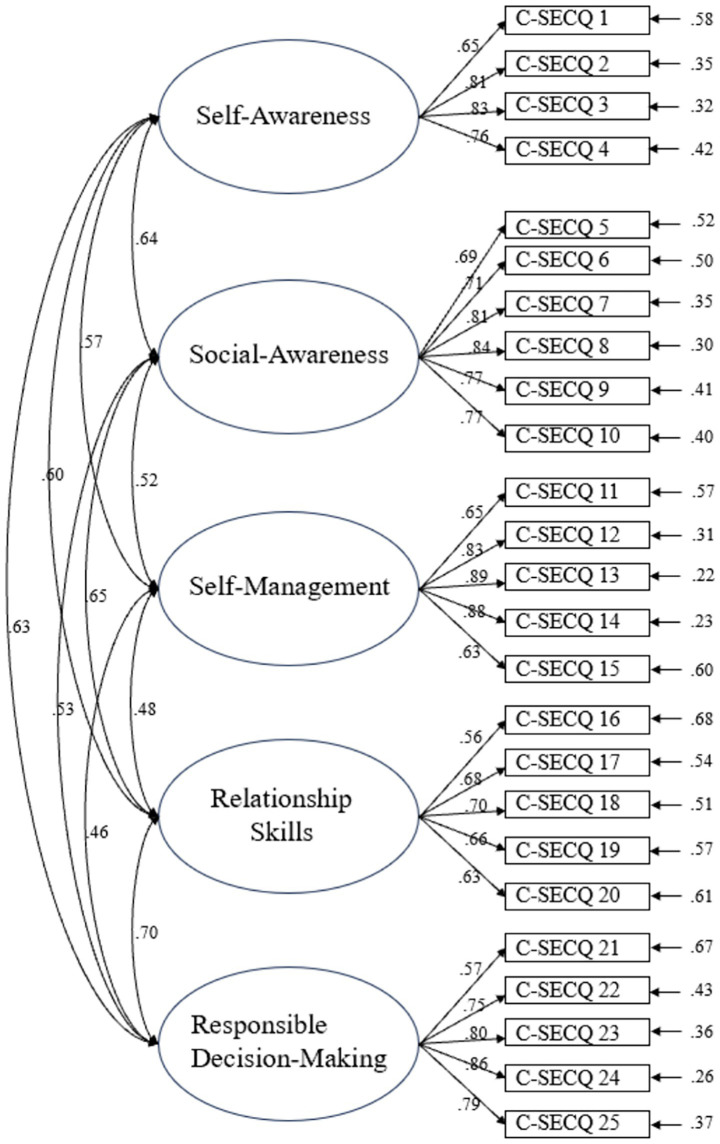
The diagram of CFA results for C-SECQ (*N* = 540). Factor labels derived from original scale structure with item 5 moved to Social Awareness.

CFA of this revised five-factor model (see Figure 5) indicated improved fit indices (CFI = 0.92, TLI = 0.91, RMSEA = 0.05, SRMR = 0.06) compared to Study 1’s original model. However, the χ^2^/*df* ratio was 16.77, which is considerably higher than conventional cutoffs (e.g., <3 or <5). While χ^2^ is sensitive to large sample sizes, this very high ratio warrants caution in interpreting overall model fit despite other favorable indices.

In terms of reliability and validity, the C-SECQ (with item 5 reassigned to Social Awareness) showed high internal consistency again (Cronbach’s *α* = 0.93). As shown in Table 5, all five factors reported good convergent (AVE > 0.50) and discriminant validity (AVE > MSV for all factors).

**Table 5:** The validity psychometric indicators of C-SECQ scale (*N* = 540).

Factor	AVE	H	ω	MSV
Self-awareness	0.59	0.85	0.80	0.41
Social awareness	0.59	0.89	0.87	0.42
Self-management	0.62	0.89	0.87	0.33
Relationship skills	0.53	0.78	0.79	0.49
Responsible decision-making	0.59	0.87	0.85	0.49

Factor labels derived from original scale structure (with item 5 moved to Social Awareness). AVE = Average Variance Extracted; H = construct replicability index; ω = McDonald’s omega; MSV = Maximum Shared Variance.

### C-SELS

**Figure 6:**
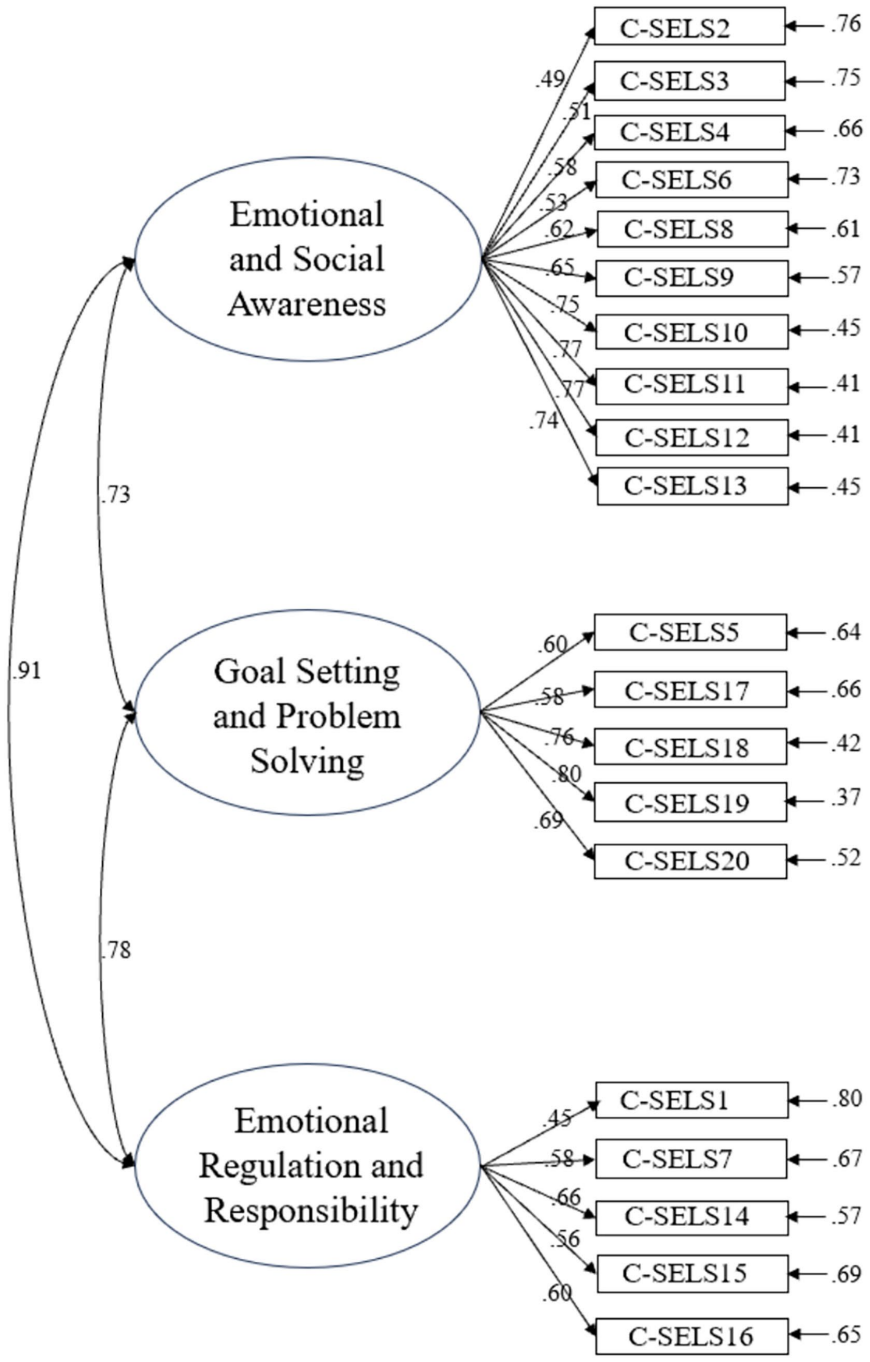
The diagram of CFA results for C-SELS (*N* = 540).

CFA (ML estimation) of this new three-factor model reported enhanced but still unsatisfactory model fit indices: CFI = 0.87, TLI = 0.85, RMSEA = 0.08, SRMR = 0.06. The χ^2^/*df* ratio was 24.51 (*χ*^2^ = 4656.33, *df =* 190), which is very high and indicates poor fit by this criterion, despite some other indices being borderline acceptable. The results of CFA are also presented in Figure 6.

These results suggest that while the C-SELS items are reliable as a whole, the proposed new three-factor structure derived from Study 1 EFA does not demonstrate adequate convergent or discriminant validity in the larger independent sample, and its model fit remains problematic.

In addition, the C-SELS showed high internal consistency in Study 2 (Cronbach’s α = 0.92). As shown in Table 6, McDonald’s *ω* coefficients for the three new C-SELS factors were acceptable, suggesting reasonable internal consistency for these new factor groupings. However, AVE values were low for “Emotional and Social Awareness” (0.44) and “Emotional Regulation and Responsibility” (0.35), indicating limited convergent validity. Furthermore, high MSV values relative to AVEs (e.g., Emotional and Social Awareness: MSV = 0.84 vs. AVE = 0.44; Emotional Regulation and Responsibility: MSV = 0.84 vs. AVE = 0.35) indicated substantial overlap and poor discriminant validity among these three factors.

**Table 6:** Validity psychometric indicators of new three-factor C-SELS model (*N* = 540).

Factor	AVE	H	ω	MSV
Emotional and social awareness	0.44	0.77	0.88	0.84
Goal setting and problem solving	0.51	0.71	0.82	0.62
Emotional regulation and responsibility	0.35	0.47	0.71	0.84

AVE = Average Variance Extracted; H = construct replicability index; ω = McDonald’s omega; MSV = Maximum Shared Variance.

## Discussion

This two-study research aimed to develop and validate Chinese versions of the Social Emotional Competence Questionnaire (C-SECQ) and the Social-Emotional Learning Scale (C-SELS) for use with Chinese university students. The results produced several significant findings: (1) C-SECQ has been successfully validated, which demonstrated high internal consistency reliability and convergent validity; (2) A new three-factor model of SEL, comprising Emotional and Social Awareness, Goal Setting and Problem Solving, and Emotional Regulation and Responsibility, emerged from the analysis of C-SELS. However, this model should be further studied because of limited convergent and discriminant validity.

The C-SECQ demonstrated strong psychometric properties across both studies. It showed high internal consistency reliability (Cronbach’s *α* and McDonald’s *ω*). After a minor adjustment suggested by EFA in Study 1 (reassigning item 5, “*I can read people’s faces when they are angry*,” from Self-Awareness to Social Awareness), the five-factor structure showed good model fit (CFI, TLI, RMSEA, SRMR) in the larger independent sample in Study 2, along with good convergent and discriminant validity (AVE > 0.50, AVE > MSV for all factors). The EFA results in Study 2 further supported this adjusted structure. The reassignment of item 5 to Social Awareness is logical, as recognizing others’ facial expressions aligns more directly with understanding others’ emotions (a facet of social awareness) than with introspective self-awareness (Borowski, 2019). This adjustment improved the psychometric profile of the Social Awareness dimension. The C-SECQ’s design, not being confined to educational settings, also suggests its potential for broader applicability. Although the χ^2^/*df* ratio for the C-SECQ in Study 2 was relatively high, this statistic is well known to be overly sensitive to large sample sizes. We therefore triangulated model adequacy using multiple fit indices (RMSEA = 0.05, CFI = 0.92, TLI = 0.91), all of which fell within recommended thresholds, supporting the overall acceptability of the model (Whittaker and Schumacker, 2022).

In terms of C-SELS, the validation proved more challenging. The original three-factor model (Task Articulation, Peer Relationships, Self-Regulation) showed poor model fit and very poor discriminant validity in Study 1. EFA in Study 1 suggested an alternative three-factor structure: Emotional and Social Awareness, Goal Setting and Problem Solving, and Emotional Regulation and Responsibility. While these new factors appeared conceptually coherent and offered interesting avenues for cultural interpretation, their psychometric performance in the independent validation sample (Study 2) was inadequate. Although internal consistency for these new factors (McDonald’s *ω*) was acceptable, the CFA model fit remained not adequate (especially considering the very high χ^2^/*df* ratio and CFI/TLI below.90), and crucial indicators of construct validity—convergent validity (low AVEs for two factors) and discriminant validity (MSV > AVE for two factors)—were not met. These sub-optimal indices suggest that certain items may benefit from further refinement or removal. Future work should experiment with shortened or rephrased versions of the scale to enhance model fit.

In spite of this, the newly proposed three-factor model of C-SELS offers valuable insights for cross-cultural studies of SEL in China. These factors can be interpreted through the lens of traditional Chinese culture and comparative education. The “Emotional and Social Awareness” factor, for example, aligns with the Confucian principle of “Do not do unto others what you do not want done to yourself” (己所不欲, 勿施于人), which emphasizes empathy and harmonious relationships. The “Goal Setting and Problem Solving” factor echoes traditional values such as “A gentleman focuses on the fundamentals” (君子务本) and “Investigate things to attain knowledge” (格物致知), emphasizing the importance of understanding core principles when pursuing goals (Eryong and Li, 2021). The “Emotional Regulation and Responsibility” factor reflects collectivist ideals, particularly the emphasis on “Self-discipline and devotion to the public good” (克己奉剬), which encourages individuals to prioritize social responsibility over personal desires. These cultural values may shape how Chinese participants interpret the items, leading to nuanced differences compared to Western interpretations. For instance, the item “*I can understand that I am responsible for my own actions*” (我理解我要为自己的行为负责) might prompt Chinese individuals to consider the broader social impact of their actions, rather than focusing solely on personal accountability. While these interpretations are speculative given the poor validity of the structure itself, they highlight the importance of considering cultural frameworks when examining SEL constructs. However, cultural relevance cannot substitute for sound psychometric properties. The current findings underscore that the C-SELS, despite its items showing overall reliability, does not yet possess a clearly validated factor structure in this context.

### Implications and limitations

This study represents a significant step in adapting and validating SEL measurement tools for the Chinese context, offering valuable resources for both academic research and practical applications. By providing reliable and culturally adapted scales, such as C-SECQ and C-SELS, this work contributes to the growing body of literature on SEL and its relevance in non-Western settings. The findings from this study also offer insights into how SEL constructs may be perceived and operationalized differently across cultures, particularly in a Chinese educational context.

However, several limitations warrant consideration and suggest avenues for future research. Firstly, our sample was confined to undergraduate students with a narrow age range, which limits the generalizability of the results. To truly capture the effectiveness and applicability of these scales, future research should involve participants from a wider range of age groups, including younger students, working adults, and possibly older populations. Expanding the sample size and diversity would enhance the external validity of the findings and provide a more comprehensive understanding of SEL across different stages of life.

Another limitation is the potential influence of social desirability bias on the participants’ responses. In a culture that places high value on social harmony and face-saving, participants might have responded in ways they believed were expected, rather than providing authentic self-assessments. Future studies could address this by employing more sophisticated data collection methods, such as anonymous surveys or implicit measures, to reduce the impact of social desirability bias. Using questions with sense of humor and explaining some details of the research are also helpful to reduce social desirability bias (Bergen and Labonté, 2020).

Thirdly, while the C-SECQ showed promising results, the C-SELS requires significant further work. The psychometric issues identified suggest that a simple translation and EFA-driven re-structuring may not be sufficient. Future efforts with the C-SELS might involve qualitative work (e.g., cognitive interviews with Chinese students on item interpretation) to understand sources of misfit, followed by item revision or development of new, culturally grounded items.

Finally, this study focused on adapting existing Western scales. While a crucial first step, the long-term advancement of SEL assessment in China will benefit from the development of original scales rooted in Chinese cultural values and educational philosophies from the outset. This could involve lexical studies and qualitative research to identify culturally salient SEL constructs and item content.

## Conclusion

This study adapted and conducted a preliminary validation of the Chinese version of the Social Emotional Competence Questionnaire (C-SECQ) and explored the psychometric properties of the Chinese version of the Social-Emotional Learning Scale (C-SELS). The C-SECQ demonstrated high internal consistency reliability and, after a minor structural refinement, showed good evidence of construct validity in Chinese university students, making it a promising tool for assessing social–emotional competence in this population. In contrast, the C-SELS, despite showing high overall internal consistency, presented significant challenges in establishing a valid factor structure. Neither the original English factor structure nor a newly EFA-derived three-factor model demonstrated adequate model fit or construct validity (specifically, convergent and discriminant validity) in the independent validation sample. Therefore, the C-SELS in its current form requires substantial further research and revision before it can be confidently used in the Chinese context.

This research contributes to the availability of SEL assessment tools for Chinese university students and underscores the critical importance of rigorous psychometric evaluation, including independent sample validation, when adapting instruments across cultures. The findings highlight the C-SECQ as a potentially valuable measure, while also illustrating the complexities and challenges inherent in cross-cultural scale adaptation.

## Data Availability

The raw data supporting the conclusions of this article will be made available by the authors, without undue reservation.
