# Supplementary material for: Development and psychometric properties of Chinese social emotional competence measurements
Source: Front Psychol. 2025 Jul 31;16:1574923. doi: 10.3389/fpsyg.2025.1574923 (PMC12351928; doi:10.3389/fpsyg.2025.1574923)
Supplement: Supplementary file 1 [file Table_1.docx]

**Appendix**

**Table S1.** *The Descriptive Statistics of each item of C-SECQ (N = 195)*

|  | Mean | Median | Skewness | Kurtosis | Range | Minimum | Maximum |
| --- | --- | --- | --- | --- | --- | --- | --- |
| C-SECQ1 | 4.59 | 5.00 | -0.94 | 1.58 | 5 | 1 | 6 |
| C-SECQ2 | 4.57 | 5.00 | -1.23 | 1.80 | 5 | 1 | 6 |
| C-SECQ3 | 4.75 | 5.00 | -1.07 | 1.61 | 5 | 1 | 6 |
| C-SECQ4 | 4.86 | 5.00 | -1.08 | 1.12 | 5 | 1 | 6 |
| C-SECQ5 | 4.81 | 5.00 | -0.92 | 1.18 | 5 | 1 | 6 |
| C-SECQ6 | 4.69 | 5.00 | -1.36 | 3.08 | 5 | 1 | 6 |
| C-SECQ7 | 4.51 | 5.00 | -0.51 | -0.16 | 5 | 1 | 6 |
| C-SECQ8 | 4.28 | 4.00 | -0.51 | -0.29 | 5 | 1 | 6 |
| C-SECQ9 | 4.52 | 5.00 | -0.81 | 0.74 | 5 | 1 | 6 |
| C-SECQ10 | 4.43 | 5.00 | -0.56 | 0.05 | 5 | 1 | 6 |
| C-SECQ11 | 3.86 | 4.00 | -0.34 | -0.64 | 5 | 1 | 6 |
| C-SECQ12 | 4.10 | 4.00 | -0.62 | -0.16 | 5 | 1 | 6 |
| C-SECQ13 | 4.04 | 4.00 | -0.45 | -0.24 | 5 | 1 | 6 |
| C-SECQ14 | 4.05 | 4.00 | -0.46 | -0.19 | 5 | 1 | 6 |
| C-SECQ15 | 4.21 | 4.00 | -0.52 | -0.20 | 5 | 1 | 6 |
| C-SECQ16 | 4.72 | 5.00 | -0.74 | 0.30 | 5 | 1 | 6 |
| C-SECQ17 | 4.90 | 5.00 | -1.07 | 1.61 | 5 | 1 | 6 |
| C-SECQ18 | 4.57 | 5.00 | -0.52 | -0.04 | 5 | 1 | 6 |
| C-SECQ19 | 4.53 | 5.00 | -0.72 | 0.69 | 5 | 1 | 6 |
| C-SECQ20 | 4.77 | 5.00 | -1.05 | 1.15 | 5 | 1 | 6 |
| C-SECQ21 | 4.86 | 5.00 | -1.06 | 1.56 | 5 | 1 | 6 |
| C-SECQ22 | 4.97 | 5.00 | -1.38 | 2.64 | 5 | 1 | 6 |
| C-SECQ23 | 4.99 | 5.00 | -1.49 | 3.23 | 5 | 1 | 6 |
| C-SECQ24 | 4.82 | 5.00 | -1.25 | 2.26 | 5 | 1 | 6 |
| C-SECQ25 | 5.04 | 5.00 | -1.32 | 2.83 | 5 | 1 | 6 |

Note. Standard Error of Skewness = .17; Standard Error of Kurtosis = .35

**Table S2.** *The Descriptive Statistics of each item of C-SELS (N = 195)*

|  | Mean | Median | Skewness | Kurtosis | Range | Minimum | Maximum |
| --- | --- | --- | --- | --- | --- | --- | --- |
| C-SELS1 | 4.19 | 4.00 | -0.98 | 2.98 | 4 | 1 | 5 |
| C-SELS2 | 3.83 | 4.00 | -0.62 | 0.20 | 4 | 1 | 5 |
| C-SELS3 | 3.81 | 4.00 | -0.88 | 0.64 | 4 | 1 | 5 |
| C-SELS4 | 3.95 | 4.00 | -1.02 | 1.65 | 4 | 1 | 5 |
| C-SELS5 | 3.96 | 4.00 | -1.18 | 2.53 | 4 | 1 | 5 |
| C-SELS6 | 3.59 | 4.00 | -0.62 | 0.24 | 4 | 1 | 5 |
| C-SELS7 | 4.08 | 4.00 | -0.96 | 2.65 | 4 | 1 | 5 |
| C-SELS8 | 4.02 | 4.00 | -1.10 | 2.50 | 4 | 1 | 5 |
| C-SELS9 | 4.04 | 4.00 | -1.12 | 1.75 | 4 | 1 | 5 |
| C-SELS10 | 3.82 | 4.00 | -0.65 | 0.25 | 4 | 1 | 5 |
| C-SELS11 | 3.90 | 4.00 | -0.79 | 0.56 | 4 | 1 | 5 |
| C-SELS12 | 3.89 | 4.00 | -0.71 | 0.94 | 4 | 1 | 5 |
| C-SELS13 | 4.01 | 4.00 | -1.06 | 2.28 | 4 | 1 | 5 |
| C-SELS14 | 4.43 | 4.00 | -1.26 | 3.51 | 4 | 1 | 5 |
| C-SELS15 | 4.14 | 4.00 | -1.36 | 3.04 | 4 | 1 | 5 |
| C-SELS16 | 4.03 | 4.00 | -1.07 | 2.19 | 4 | 1 | 5 |
| C-SELS17 | 3.87 | 4.00 | -0.83 | 0.83 | 4 | 1 | 5 |
| C-SELS18 | 3.76 | 4.00 | -0.92 | 1.23 | 4 | 1 | 5 |
| C-SELS19 | 3.89 | 4.00 | -0.98 | 1.43 | 4 | 1 | 5 |
| C-SELS20 | 3.88 | 4.00 | -1.07 | 1.74 | 4 | 1 | 5 |

Note. Standard Error of Skewness = .17; Standard Error of Kurtosis = .35

**Table S3.** *Correlational Matrix for Dimensions of C-SECQ and C-SELS (Study 1, N=195)*

|  |  | 1 | 2 | 3 | 4 | 5 | 6 | 7 | 8 |
| --- | --- | --- | --- | --- | --- | --- | --- | --- | --- |
| 1 | C-SECQ_Self-Awareness |  |  |  |  |  |  |  |  |
| 2 | C-SECQ_Social Awareness | .713^**^ |  |  |  |  |  |  |  |
| 3 | C-SECQ_Self-Management | .473^**^ | .486^**^ |  |  |  |  |  |  |
| 4 | C-SECQ_Relationship Management | .530^**^ | .535^**^ | .403^**^ |  |  |  |  |  |
| 5 | C-SECQ_Responsible Decision-Making | .536^**^ | .456^**^ | .438^**^ | .527^**^ |  |  |  |  |
| 6 | C-SELS_Task Articulation | .661^**^ | .555^**^ | .453^**^ | .547^**^ | .470^**^ |  |  |  |
| 7 | C-SELS_Peer Relationship | .631^**^ | .688^**^ | .421^**^ | .625^**^ | .566^**^ | .783^**^ |  |  |
| 8 | C-SELS_Self Regulation | .607^**^ | .497^**^ | .451^**^ | .558^**^ | .550^**^ | .757^**^ | .769^**^ |  |

Note. **. Correlation is significant at the 0.01 level (2-tailed).

**Table S4.** *The Descriptive Statistics of each item of C-SECQ (N = 540)*

|  | Mean | Median | Skewness | Kurtosis | Range | Minimum | Maximum |
| --- | --- | --- | --- | --- | --- | --- | --- |
| C-SECQ1 | 4.48 | 5.00 | -0.77 | 0.64 | 5 | 1 | 6 |
| C-SECQ2 | 4.60 | 5.00 | -0.78 | 1.05 | 5 | 1 | 6 |
| C-SECQ3 | 4.67 | 5.00 | -0.72 | 0.97 | 5 | 1 | 6 |
| C-SECQ4 | 4.80 | 5.00 | -0.80 | 0.85 | 5 | 1 | 6 |
| C-SECQ5 | 4.76 | 5.00 | -0.66 | 0.66 | 5 | 1 | 6 |
| C-SECQ6 | 4.71 | 5.00 | -1.10 | 2.36 | 5 | 1 | 6 |
| C-SECQ7 | 4.61 | 5.00 | -0.47 | -0.15 | 5 | 1 | 6 |
| C-SECQ8 | 4.40 | 5.00 | -0.62 | 0.16 | 5 | 1 | 6 |
| C-SECQ9 | 4.40 | 5.00 | -0.74 | 0.49 | 5 | 1 | 6 |
| C-SECQ10 | 4.40 | 5.00 | -0.63 | 0.32 | 5 | 1 | 6 |
| C-SECQ11 | 4.20 | 4.00 | -0.54 | -0.07 | 5 | 1 | 6 |
| C-SECQ12 | 4.11 | 4.00 | -0.50 | -0.11 | 5 | 1 | 6 |
| C-SECQ13 | 4.13 | 4.00 | -0.43 | -0.23 | 5 | 1 | 6 |
| C-SECQ14 | 4.13 | 4.00 | -0.48 | 0.13 | 5 | 1 | 6 |
| C-SECQ15 | 4.18 | 4.00 | -0.45 | -0.14 | 5 | 1 | 6 |
| C-SECQ16 | 4.53 | 5.00 | -0.62 | 0.18 | 5 | 1 | 6 |
| C-SECQ17 | 4.81 | 5.00 | -0.81 | 0.90 | 5 | 1 | 6 |
| C-SECQ18 | 4.63 | 5.00 | -0.59 | 0.54 | 5 | 1 | 6 |
| C-SECQ19 | 4.57 | 5.00 | -0.68 | 0.78 | 5 | 1 | 6 |
| C-SECQ20 | 4.64 | 5.00 | -0.71 | 0.37 | 5 | 1 | 6 |
| C-SECQ21 | 4.95 | 5.00 | -0.90 | 1.01 | 5 | 1 | 6 |
| C-SECQ22 | 4.88 | 5.00 | -1.05 | 1.81 | 5 | 1 | 6 |
| C-SECQ23 | 4.95 | 5.00 | -0.95 | 1.32 | 5 | 1 | 6 |
| C-SECQ24 | 4.90 | 5.00 | -0.88 | 1.57 | 5 | 1 | 6 |
| C-SECQ25 | 4.96 | 5.00 | -0.81 | 1.55 | 5 | 1 | 6 |

Note. Standard Error of Skewness = .11; Standard Error of Kurtosis = .21

**Table S5.** *The Descriptive Statistics of each item of C-SELS (N = 540)*

|  | Mean | Median | Skewness | Kurtosis | Range | Minimum | Maximum |
| --- | --- | --- | --- | --- | --- | --- | --- |
| C-SELS1 | 4.59 | 5.00 | -0.42 | 1.12 | 4 | 1 | 5 |
| C-SELS2 | 3.97 | 4.00 | -0.70 | 0.66 | 4 | 1 | 5 |
| C-SELS3 | 3.73 | 4.00 | -0.76 | 0.19 | 4 | 1 | 5 |
| C-SELS4 | 3.78 | 4.00 | -0.82 | 0.38 | 4 | 1 | 5 |
| C-SELS5 | 3.95 | 4.00 | -0.98 | 1.74 | 4 | 1 | 5 |
| C-SELS6 | 3.76 | 4.00 | -0.86 | 0.87 | 4 | 1 | 5 |
| C-SELS7 | 3.83 | 4.00 | -0.83 | 0.87 | 4 | 1 | 5 |
| C-SELS8 | 4.00 | 4.00 | -0.84 | 1.36 | 4 | 1 | 5 |
| C-SELS9 | 4.05 | 4.00 | -1.03 | 1.68 | 4 | 1 | 5 |
| C-SELS10 | 3.91 | 4.00 | -0.80 | 1.05 | 4 | 1 | 5 |
| C-SELS11 | 3.87 | 4.00 | -0.75 | 0.64 | 4 | 1 | 5 |
| C-SELS12 | 3.83 | 4.00 | -0.74 | 0.94 | 4 | 1 | 5 |
| C-SELS13 | 3.95 | 4.00 | -1.12 | 2.52 | 4 | 1 | 5 |
| C-SELS14 | 4.21 | 4.00 | -0.91 | 1.66 | 4 | 1 | 5 |
| C-SELS15 | 4.25 | 4.00 | -1.25 | 2.86 | 4 | 1 | 5 |
| C-SELS16 | 4.08 | 4.00 | -1.00 | 1.71 | 4 | 1 | 5 |
| C-SELS17 | 3.99 | 4.00 | -0.83 | 1.15 | 4 | 1 | 5 |
| C-SELS18 | 3.84 | 4.00 | -0.76 | 0.69 | 4 | 1 | 5 |
| C-SELS19 | 3.82 | 4.00 | -0.75 | 0.65 | 4 | 1 | 5 |
| C-SELS20 | 3.86 | 4.00 | -0.91 | 1.15 | 4 | 1 | 5 |

Note. Standard Error of Skewness = .11; Standard Error of Kurtosis = .21

**Table S6.** *Correlational Matrix for Dimensions of C-SECQ and C-SELS (Study 2, N=540)*

|  |  | 1 | 2 | 3 | 4 | 5 | 6 | 7 | 8 |
| --- | --- | --- | --- | --- | --- | --- | --- | --- | --- |
| 1 | C-SECQ_Self-Awareness |  |  |  |  |  |  |  |  |
| 2 | C-SECQ_Social Awareness | .565^**^ |  |  |  |  |  |  |  |
| 3 | C-SECQ_Self-Management | .513^**^ | .499^**^ |  |  |  |  |  |  |
| 4 | C-SECQ_Relationship Management | .487^**^ | .551^**^ | .447^**^ |  |  |  |  |  |
| 5 | C-SECQ_Responsible Decision-Making | .558^**^ | .488^**^ | .444^**^ | .604^**^ |  |  |  |  |
| 6 | C-SELS_Emotional and Social Awareness | .602^**^ | .648^**^ | .482^**^ | .587^**^ | .519^**^ |  |  |  |
| 7 | C-SELS_Goal Setting and Problem Solving | .576^**^ | .500^**^ | .491^**^ | .456^**^ | .461^**^ | .671^**^ |  |  |
| 8 | C-SELS_Emotional Regulation and Responsibility | .581^**^ | .544^**^ | .383^**^ | .597^**^ | .586^**^ | .732^**^ | .623^**^ |  |

Note. **. Correlation is significant at the 0.01 level (2-tailed).
